# Lubrication Performance of Graphene as Lubricant Additive in 4-n-pentyl-4′-cyanobiphyl Liquid Crystal (5CB) for Steel/Steel Contacts

**DOI:** 10.3390/ma11112110

**Published:** 2018-10-26

**Authors:** Zhiliang Li, Chonghai Xu, Guangchun Xiao, Jingjie Zhang, Zhaoqiang Chen, Mingdong Yi

**Affiliations:** 1 School of Mechanical and Automotive Engineering, Qilu University of Technology (Shandong Academy of Sciences), Jinan 250353, China; 18396814380@163.com (Z.L.); xgc@qlu.edu.cn (G.X.); zjj@qlu.edu.cn (J.Z.); czq@qlu.edu.cn (Z.C.); new-raul@163.com (M.Y.); 2 Key Laboratory of Equipments Manufacturing and Intelligent Measurement and Control, China National Light Industry, Qilu University of Technology (Shandong Academy of Sciences), Jinan 250353, China

**Keywords:** graphene, 5CB, lubricant additive, lubrication performance

## Abstract

The lubrication performance of graphene used as additive in 4-n-pentyl-4′-cyanobiphyl liquid crystal (5CB) for steel/steel contacts was studied on a ball-on-plate tribotester. The friction test results show that when the graphene content in the 5CB was 0.15 wt.%, and the lubricant and friction pairs were heated to 44–46 °C before friction tests, the lubrication performance of the 5CB was most improved. Compared with pure 5CB, 5CB+0.15 wt.% graphene suspension reduced the friction coefficient and wear scar diameter by up to 70.6% and 41.3%, respectively. The lubrication mechanisms have been tentatively proposed according to the test results. We speculate that the excellent lubrication performance of graphene/5CB suspensions may be attributed to the low shear resistance adsorption layer formed by graphene and 5CB molecules on the sliding surfaces. As the protective layer, it not only prevents direct contact between the rough sliding surfaces but also is easy to slide.

## 1. Introduction

Friction and wear between mechanical parts widely exist in mechanical systems. Friction consumes a lot of energy, and wear will lead to premature failure of parts [1]. In order to improve the service efficiency and life of mechanical equipment, friction and wear between parts must be reduced. Lubrication technology is the key technology to solve friction and wear and prolong the service life of equipment. Lubricants are widely used in industry to protect products and machinery equipment from wear. In addition, the application of lubricants also reduces the friction coefficient of the manufacturing process and eliminates excess heat accumulated in the mechanical system. Therefore, improving lubricant performance is important for protecting machinery from severe damage and reducing energy consumption [2].

Liquid crystals exhibit mesophase between liquid and crystal phase, which have both crystal anisotropy and liquid fluidity. Liquid crystals can be classified in two categories: (1) monomer liquid crystals, irrespective of whether they are able to polymerize, and (2) polymer liquid crystals [3]. 5CB belongs to monomer liquid crystals. When liquid crystals are used as lubricants, their molecules exhibit a crystal characteristic with a strong carrying capacity in the direction perpendicular to the friction surface. In the sliding shear direction, the liquid crystal exhibits a low viscosity liquid characteristic with a low friction coefficient [4]. It is believed that the liquid crystal with nematic phase (internal molecules aligned in one direction) has the best lubricity. The molecule of 5CB consists of a rigid biphenyl/cyano backbone with a flexible alkyl chain and presents a nematic phase at room temperature [5,6]. 5CB is expected to be widely used in industrial production due to its high bearing capacity and low shear resistance when used as lubricant.

Recently, nanoparticles have been introduced into industry as a new lubricant additive [7,8,9]. The performance of some lubricants can be significantly improved by the addition of suitable nanoparticle additives, especially some flake nanoparticle additives. Among various flaky nanoparticles, graphene and graphene oxide (GO) have attracted worldwide attention since their discovery [10,11]. The unique sheet structure makes graphene and GO easily enter the friction contact surfaces. Graphene and GO have strong mechanical properties and stable chemical properties [12]. So they have excellent antiwear properties and may become effective lubricant additives [13,14]. In recent decades, many studies have been conducted on the lubricating properties of graphene and GO [15].

Multilayer graphene can be well dispersed in some specific lubricants, and shows its antifriction and antiwear properties. For instance, Fan et al. [16] reported that the multilayer graphene can be well dispersed in bentonite grease and provides lower friction and wear compared with graphite. This antifriction ability mainly depends on the lubricating films formed on the friction surfaces. Guo et al. [17] prepared multilayer graphene by supercritical CO_2_ stripping of graphite. The antifriction and antiwear properties of the multilayer graphene as an additive of polyalphaolefin-2 (PAO2) oil were studied by using four-ball test method. They pointed out that the PAO2 with 0.05 wt.% multilayer graphene showed the best lubrication performance. By analyzing the worn surfaces, it was found that the excellent lubrication performance of the multilayer graphene additives can be attributed to its small sheet diameter and layered structure, which make the multilayer graphene easily enter the friction interfaces and form a protective layer, thus preventing the friction surfaces from directly contacting.

Besides multilayer graphene, few-layer graphene also has been reported as lubricant additive. For instance, Eswaraiah et al. [18] evaluated the lubricating properties of ultra-thin graphene. The results showed that the friction coefficient and the wear scar diameter of the steel ball were reduced by 80% and 33%, respectively, at the optimum graphene concentration. Xie et al. [19] evaluated the lubrication performance of graphene used as additive in water. It was reported that graphene played a positive role in reducing friction and wear. They pointed out that the water added with 0.5 wt.% graphene showed best lubrication performance. Berman et al. [20] reported that the friction surfaces covered with the few-layer graphene significantly reduced friction and wear on steel/steel contacts. Restuccia et al. [21] reported that graphene can effectively lubricate steel-steel sliding contact. It was also confirmed that the lubrication mechanism of graphene is that the metal surfaces covered by graphene become almost inert and show very low adhesion and shear strength when mated in a sliding contact. Marchetto et al. [22] reported that graphene can significantly reduce the friction coefficient of steel-steel sliding contact. Raman spectroscopy results proved that graphene reduced the adhesion of the iron interface by making the surfaces inert, which further reduced the coefficient of friction.

To exert the lubricating properties of graphene in a lubricant, the dispersibility of graphene in the lubricant must be considered. The dispersity of graphene in most lubricants is very poor [23]. Chemical modification is usually used to improve its dispersity. For instance, Lin et al. [24] reported that after chemical modification by stearic acid and oleic acid, the dispersibility of graphene in base oil was obviously improved. The lubrication performances of the modified graphene lubricant were studied. The results showed that the addition of 0.075 wt.% of modified graphene can obviously enhance the antiwear and bearing capacity of the oil. Gupta et al. [25] added functionalized aged reduced GO additives to polyethylene glycol. The results showed that adding functionally aged reduced GO significantly improved the lubricating performance of steel-steel contact. The study found that the main reason for improving the lubricating performance was that many oxygen functional groups in the reduced GO structure after aging promoted its effective dispersion in polyethylene glycol. Choudhary et al. [26] prepared alkyl amine covalently modified GO, which was easily dispersed in nonpolar organic solvents (especially aliphatic hydrocarbon solvents). By analyzing the friction test results, it was found that when alkyl amine covalently modified GO was added to n-hexadecane lubricating oil at the optimum concentration, the friction coefficient and wear scar diameter of n-hexadecane lubricating oil were reduced by 26% and 9%, respectively, compared with the base oil. Zhang et al. [27] used oleic acid to modify graphene and dispersed it as an additive in the polyalphaolefin lubricating oil. The lubrication performances of the graphene oil were studied. When the concentration of graphene is 0.02–0.06 wt.%, the friction coefficient and the wear scar diameter were reduced by 17% and 14%, respectively, compared with the pure polyalphaolefin lubricating oil. Bordignon et al. [28] reported that the plasma functionalization of multilayer graphene can make it be well dispersed in polyolester oil and provides lower friction coefficient and lower wear loss compared with pure polyolester oil.

In addition to using chemical modification to improve the dispersion of graphene, it has also been reported to improve dispersion by changing the shape of graphene to prevent adsorption and agglomeration between sheets. Inspired by the fact that crumpled paper sheets are not easily adhered to each other, Dou et al. [29] used capillary compression to quickly evaporate aerosol droplets of GO flakes to produce crumpled graphene balls. Experiments showed that graphene balls can be stably dispersed in polyalphaolefins type 4 (PAO4) oil without obvious aggregation. The lubrication performances of the graphene balls in PAO4 oil were studied by using a pin-disk friction meter. Compared with PAO4 oil, the friction coefficient and wear coefficient with graphene balls were reduced by about 20% and 85% respectively, mainly due to their anti-aggregation properties.

The dispersion effect of graphene in base oil will directly affect its lubrication performance. However, using chemical modification to improve the dispersion of graphene will affect its chemical properties. At the same time, changing the morphology of graphene through external force to improve its dispersion will destroy the physical structure of graphene.

In order to achieve the good dispersion of graphene in lubricating oil without changing its physical and chemical properties, we chose 5CB as the basic lubricant in this study. Since the rod-shaped molecules of 5CB tend to be arranged along the surface of the nanostructure that graphene provides [30,31,32], the alternate positions on the hexagons within the graphene surface and the alkyl chains of 5CB geometrically match well [33]. Therefore, when graphene is added to 5CB, 5CB molecules will be adsorbed on graphene [34]. However, few studies have been carried out to achieve the good dispersion of graphene and improve the lubricating performance of lubricants by using the adsorption between lubricating molecules and graphene.

In this study, the adsorption between the 5CB molecules and graphene was fully utilized. Considering the fact that the higher the temperature, the more intense the thermal movement of the liquid molecules and the more fierce the Brownian movement, we have achieved good dispersion of graphene in 5CB by heating the graphene/5CB suspension. The lubrication properties of the suspension were tested on a tribometer with a ball-on-plate configuration. Transmission electron microscopy (TEM), optical microscopy, scanning electron microscopy (SEM), energy dispersive spectroscopy (EDS) and Raman spectroscopy were used to characterize graphene and study the wear surfaces. Based on the above tests and analysis results, we deduced the possible antifriction and antiwear mechanism of graphene as additive in 5CB.

## 2. Experimental

### 2.1. Materials

The 5CB was supplied by the Shijiazhuang Huarui Scientific and Technological Co. (Shijiazhuang, China). This liquid crystal exhibits a nematic phase at 22–35 °C [5,6]. The graphene was provided by Pioneer Nanotechnology Co. (Nanjing, China) with the specification of 0.2–2 μm in sheet diameter and 0.8–1.2 nm in thickness. The anhydrous ethanol and acetone were purchased from Tianjin Guangfu Technology Development Co. (Tianjin, China).

### 2.2. Preparation of Graphene/5CB Suspension

The preparation procedures of Graphene/5CB suspension are as follows: Firstly, 5 mg of graphene was added in 10 ml of anhydrous ethanol with ultrasonic dispersion for 1 h to ensure that graphene was uniformly dispersed the solution. Secondly, 10 g of 5CB was added to graphene/anhydrous ethanol solution in 40–50 °C water bath with ultrasonication for 1 h to obtain a homogeneous suspension. After that, the suspension was put in vacuum at 50 °C for 12 h to completely evaporate the anhydrous ethanol. Finally the suspension of 0.05 wt.% graphene in 5CB (5CB+0.05 wt.% G suspension) was obtained. According to the above method, 5CB+0.15 wt.% G suspension, 5CB+0.25 wt.% G suspension and 5CB+0.35 wt.% G suspension were prepared respectively. No additional dispersion or surfactant was used to improve the dispersion of graphene in 5CB.

### 2.3. Friction Tests

**Figure 1:**
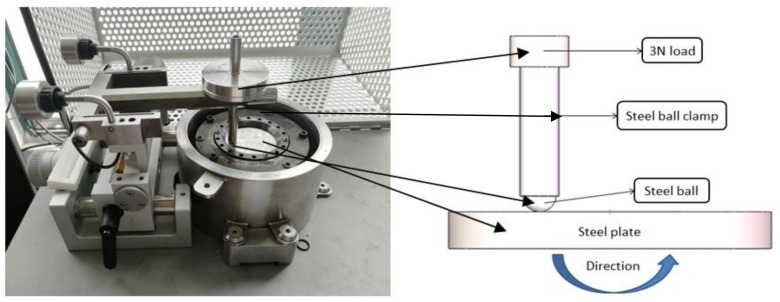
The high temperature tribometer and schematic view of ball-on-plate assembly.

The friction tests were carried out on a high temperature tribometer (Anton Paar, Buchs, Switzerland) with a ball-on-plate configuration, in indoor air conditions (24–26 °C, 37–46% RH). The tribometer is shown in Figure 1. Steel balls and plates were all made of GCr15 steel. The diameter of the steel ball was 6 mm, with roughness Ra = 25 nm. The steel plate with 55 mm in diameter, 12 mm in thickness and roughness Ra < 110 nm was used as counter-sample.

Before conducting the friction tests, the steel balls and steel plates were ultrasonically washed with acetone, washed with anhydrous ethanol and dried. During the tests, the steel plate rotated at a speed of 200 rpm and the friction track radius was 18 mm, which was equivalent to sliding at a speed of 0.38 ms^−1^. The normal load applied to the steel ball was 3 N. The duration for each sliding friction test was 1 h.

It is well known that the concentration of additives in basic lubricants plays an important role in reducing friction and wear [35]. In order to investigate the influence of graphene concentration on lubrication performance, graphene/5CB suspensions with graphene concentrations of 0 wt.%, 0.05 wt.%, 0.15 wt.%, 0.25 wt.%, and 0.35 wt.% were investigated, respectively.

For the influence of temperature on lubrication performance of the graphene/5CB suspension, three different temperature conditions were investigated. The first temperature condition: the steel plate was heated to 44–46 °C while the graphene/5CB suspension lubricant was stirred at 44–46 °C for 120 s, to achieve good dispersion of the graphene in the 5CB, then the graphene/5CB suspension lubricant was applied to the plate and immediately stopping heating at the beginning of the friction test. The heating was stopped at the beginning of the friction test to make the 5CB return to the nematic phase, thus restoring the lubrication properties of the 5CB as a liquid crystal lubricant. After the friction test started, the shear force caused the graphene to be uniformly distributed on the surface of the friction region. Therefore, it is not necessary to rely on continuous heating to maintain a good dispersion state of graphene. The second temperature condition was at room temperature (24–26 °C). The third temperature condition was at 44–46 °C.

Before the friction tests, the lubricant was dripped onto the steel plate with a dropper. Then the wear track surface was fully covered with a lubricating layer formed by the lubricant droplets. Each test condition was repeated at least three times, and the final results of friction coefficient and wear scar diameter were the average values.

### 2.4. Surface Analyses

Before analyzing the friction surfaces, the steel balls and steel plates were washed with anhydrous ethanol to remove contaminants. An optical microscope (VHX-5000, Keyence, Osaka, Japan) was used to observe wear profile and measure diameter of wear scar. Scanning electron microscopy (SEM, JEM-5600 LV, JEOL, Tokyo, Japan) was used to further analyze the surface textures of steel ball wear scar. Energy dispersive spectroscopy (EDS) was used to analyze the content of various elements on the wear surfaces. Raman microscope (Renishaw, London, UK) was used to study Raman spectra of the graphene and wear scar. The transmission electron microscope (TEM, JEM-2100, JEOL Ltd, Tokyo, Japan) was used to analyze the structure and morphology of graphene before and after the friction tests.

## 3. Results and Discussion

### 3.1. Influence of Graphene Concentration on Lubrication Performance

In order to achieve good dispersion of graphene in the 5CB and make sure the 5CB exhibits a nematic phase during the rubbing process, the friction tests were performed in the first temperature condition. The friction coefficient and the wear scar diameter at different graphene concentrations were studied.

**Figure 2:**
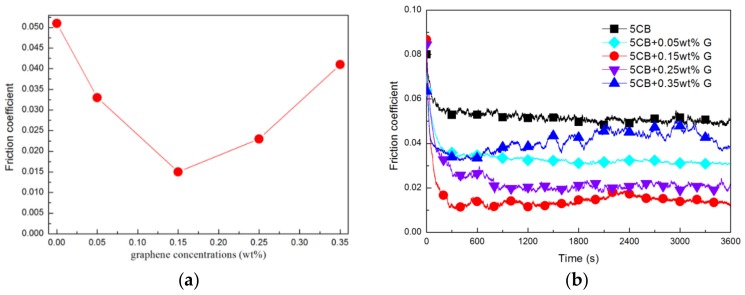
(**a**) Relationship between average friction coefficient and graphene concentration in 5CB, (**b**) relationship between friction coefficient and time at various graphene concentrations.

The relationship between the concentration of graphene in 5CB and the average friction coefficient is shown in Figure 2a. Clearly, compared with pure 5CB (μ = 0.051), all the graphene/5CB suspensions show lower friction coefficient. The coefficient of friction decreases as the concentration of graphene increases, when the addition amount is less than 0.15 wt.%. However, as the concentration increases, the friction coefficient increases, when the graphene concentration is higher than 0.15 wt.%. So the lowest value of friction coefficient (μ = 0.015, about 70.6% less than that of pure 5CB) is achieved when the graphene concentration is 0.15 wt.%.

The relationship between the friction coefficient and the time is shown in Figure 2b. It can be found that in the first 300 s, the friction coefficient of all lubricants decreased significantly with time. All the graphene/5CB suspensions show a greater reduction in friction coefficient than pure 5CB. Observing the temperature change of the steel plate, it was found that when the friction test was carried out for 300 s, the temperature of the steel plate decreased to room temperature (24–26 °C). This indicates that the friction coefficient decreases and tends to be stable during the phase transition of 5CB to nematic phase.

When the addition amount of graphene is less than 0.15 wt.%, the coefficient of friction of the lubricant is relatively stable, this may be attributed to the absence of excess graphene on the friction surfaces and all graphene is adsorbed on the friction surfaces to fill surface pits or grooves to make the friction surfaces smooth [36,37]. When the addition amount of graphene exceeds 0.15 wt.%, the friction coefficient shows a clear fluctuation and increases with the increase of addition amount. Especially when the addition amount reaches 0.35 wt.%, the friction coefficient fluctuates violently with time, and the average friction coefficient is also large (μ = 0.041). This phenomenon indicates that the concentration of added graphene is too large and has affected the lubrication effect. This may be due to the fact that graphene tends to agglomerate into large bodies at higher concentrations, and it is not easy to form a continuous and uniform antifriction layer on the friction region.

Graphene, as a sheet-like nano material, is easy to enter the friction region [38]. In addition, graphene sheets with weak van der waals force are easy to slide between each other [39,40]. So we speculate that the addition of graphene reduces the friction coefficient.

**Figure 3:**
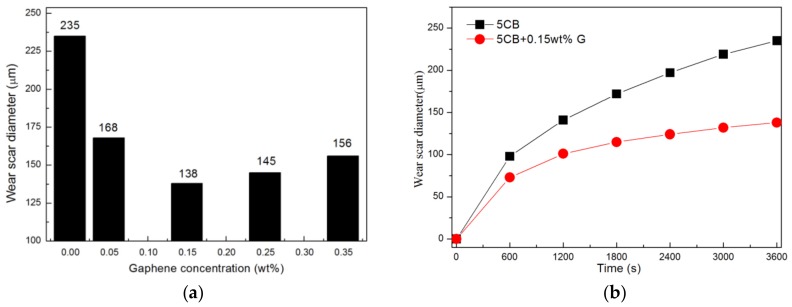
(**a**) The average wear scar diameter at various graphene concentrations after friction tests. (**b**) The average wear scar diameter of 5CB and 5CB+0.15 wt.% G lubricants at different times during friction tests.

Figure 3a presents the average wear scar diameter of the balls at various graphene concentrations. Clearly, all the graphene/5CB suspensions show smaller wear scar diameter, compared with pure 5CB (d = 235 μm). The lowest value of wear scar diameter (d = 138 μm, about 41.3% less than that of pure 5CB) is achieved when the graphene concentration is 0.15 wt.%.

When addition amount of graphene is less than 0.15 wt.%, the average diameter of steel ball wear scar decreases with the increase of concentration. This phenomenon further proves that when the concentration is less than 0.15 wt.%, there is not enough graphene to form an antifriction and antiwear protective layer covering the wear surface [37]. The contact surfaces of the friction pairs cannot be completely separated, resulting in a larger friction coefficient and a larger wear scar diameter.

However, when the graphene concentration is higher than 0.15 wt.%, the average wear scar diameter starts to increase. This phenomenon further proves that graphene tends to agglomerate into large bodies at higher concentrations, and it is not easy to form a continuous and uniform antifriction layer on the friction region [41,42]. In addition, excessive graphene on the surface affects the formation of protective layer with low shear stress and self-repair [43].

Figure 3b shows a graph of steel balls wear scar diameter versus friction time. The antiwear mechanism of graphene can be inferred by analyzing the changes of wear scar diameter of balls with friction time under the lubrication conditions of 5CB and 5CB+0.15 wt.% G, respectively. As shown in Figure 3b, from the beginning of the friction tests, the wear scar diameter of the balls lubricated by 5CB+0.15 wt.% G suspension is smaller than that lubricated by 5CB in the same period of time. In addition, it can also be found that the wear scar diameter of balls increases steadily with time under the lubrication of 5CB. While the wear scar diameter of balls increases relatively slowly with time under the lubrication of 5CB+0.15 wt.% G suspension, especially after 1200 s of test, the average wear scar diameter increases very slowly. It can be speculated that 5CB+0.15 wt.% G suspension formed a protective layer at the friction track during the friction tests, which effectively prevented the direct contact between the two contact surfaces [41,44]. Therefore, compared with 5CB lubrication, 5CB+0.15 wt.% G suspension exhibits more excellent antiwear properties.

### 3.2. Influence of Temperature on Lubrication Performance

**Figure 4:**
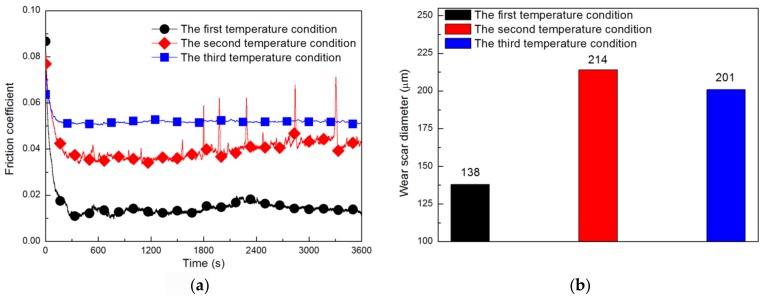
Friction coefficient curves (**a**) and the average wear scar diameters (**b**) of 5CB+0.15 wt.% G suspension under three temperature conditions.

The friction coefficient and wear scar diameter of the 5CB+0.15 wt.% G suspension were tested under the three temperature conditions set. The variation of the friction coefficient with the friction test time is shown in Figure 4a. As can be seen from Figure 4a, under the first temperature condition, the friction coefficient value is the lowest (μ = 0.015) and is relatively stable during the whole test process. This can be attributed to the good dispersion of graphene at the first temperature and the phase state of 5CB returning to nematic phase. Under the second temperature condition, the friction coefficient increased to 0.041 and there were many fluctuations during the tests, which might be the result of uneven graphene dispersion (under the second temperature condition, the friction coefficient of pure 5CB is 0.057). Under the third temperature condition, the coefficient of friction is the highest (μ = 0.052), which can be attributed to the change of phase of 5CB under this temperature condition, which damages its lubrication performance as a liquid crystal [45,46] (under the third temperature condition, the friction coefficient of pure 5CB is 0.071).

Figure 4b shows the average wear scar diameter of balls under the three temperature conditions after the friction tests. At the first temperature, the average wear scar diameter of the balls is the smallest (d = 138 μm), which may be because the well dispersed graphene forms a complete protective layer on the friction surfaces, preventing the friction surfaces from directly contacting, thus reducing wear. Under the second temperature condition, the average wear scar diameter of the ball is the largest (d = 214 μm), which may be because the uneven graphene on the friction surface not only cannot form a complete protective layer but also acts as abrasive particles to destroy the lubricating film. Therefore, the wear of steel balls cannot be effectively reduced (under the second temperature condition, the average wear scar diameter of ball lubricated by pure 5CB was 203 μm). Under the third temperature condition, the average wear scar diameter of the ball is also very large (d = 201 μm), which may be because the 5CB no longer has the lubricating properties of liquid crystal [45,46], thus increasing the average wear scar diameter of the balls (under the third temperature condition, the average wear scar diameter of steel ball lubricated by pure 5CB was 271 μm). The analysis of the average wear scar diameter of the steel balls shown in Figure 4b supports the previous assumptions about the friction and antiwear mechanism of graphene.

### 3.3. Analysis and Discussion on Lubrication Mechanism

The lubrication mechanism of 5CB+0.15 wt.% G suspension were emphatically analyzed with 5CB as the benchmark. All the test data and images analyzed were obtained under the first temperature condition.

#### 3.3.1. Wear Surface Morphology of Steel Balls under Optical Microscope

**Figure 5:**
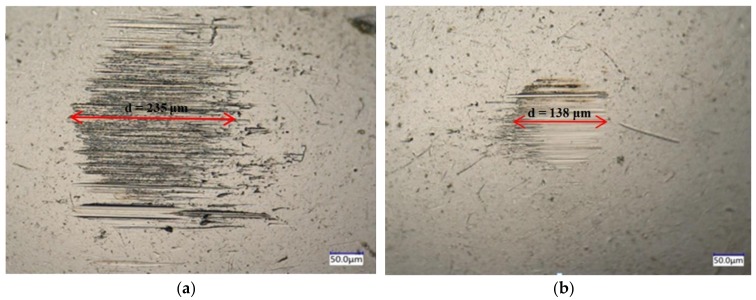
Optical micrographs of wear scars of the steel balls lubricated with (**a**) 5CB and (**b**) 5CB+0.15 wt.% G.

To determine how graphene influences lubrication performance, the topography of wear surfaces after tests were investigated. As shown in Figure 5, the wear scar diameter of the ball lubricated with 5CB+0.15 wt.% G was much smaller than that of the ball lubricated with 5CB. The wear scar diameter of the ball for 5CB was very large, and the wear surface was very rough with irregular and deep grooves, as shown in Figure 5a. On the contrary, the wear scar diameter of the ball under 5CB+0.15 wt.% G lubrication was very small, and the worn surface was neat with even distribution of relatively shallow furrows, as shown in Figure 5b. This may be related to graphene adsorbing on and passivating the friction surfaces, because some literatures believe that graphene can reduce friction and wear in steel by making the surface inert [21,22]. By analyzing the wear scar morphology, it can be found that the addition of graphene reduces the wear scar diameter and roughness.

#### 3.3.2. SEM and EDS Analysis

**Figure 6:**
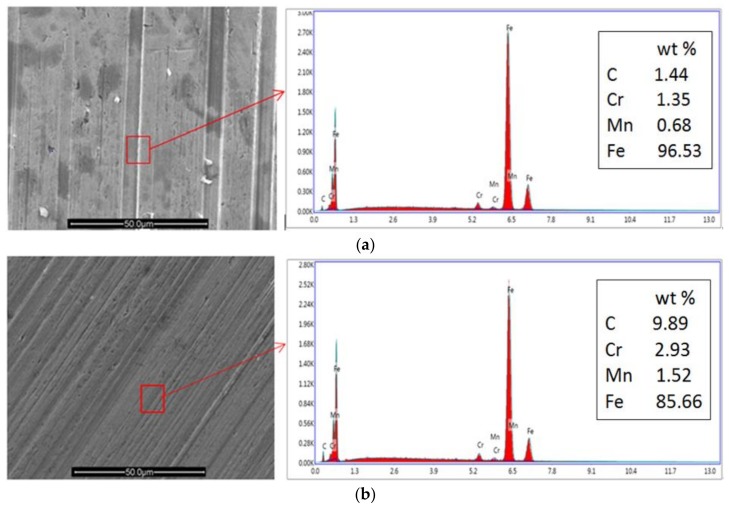
SEM micrographs of the friction surfaces of the ball and the corresponding energy dispersive spectroscopy analysis: (**a**) Lubricated with 5CB; (**b**) lubricated with 5CB+0.15 wt.% G suspension.

In order to prove that graphene exists on the friction contact surfaces and plays an important role in the friction tests process, the wear surfaces of the balls lubricated by 5CB and 5CB+0.15 wt.% G suspension were analyzed by SEM and EDS, respectively. The test results are shown in Figure 6.

Obviously, the friction surface of the ball for 5CB had irregular and deep furrows, and the severe scratch was attributed to the relatively poor antiwear property of 5CB. The corresponding EDS analysis showed that the content of C element on the worn surface was only 1.44 wt.%, which may be due to the basal component of the steel ball and carbonizations of the 5CB, as shown in Figure 6a.

By contrast, some very regular and shallow furrows appeared on the wear surface of the ball for 5CB+0.15 wt.% G suspension. Many literatures show that friction coefficient is closely related to surface morphology [47,48]. In addition, the corresponding EDS analysis showed that the C element content on the worn surface was up to 9.89 wt.%, as shown in Figure 6b. Since graphene is composed of carbon, we can infer that graphene exists on the worn surface.

#### 3.3.3. Raman Spectroscopy Analysis

**Figure 7:**
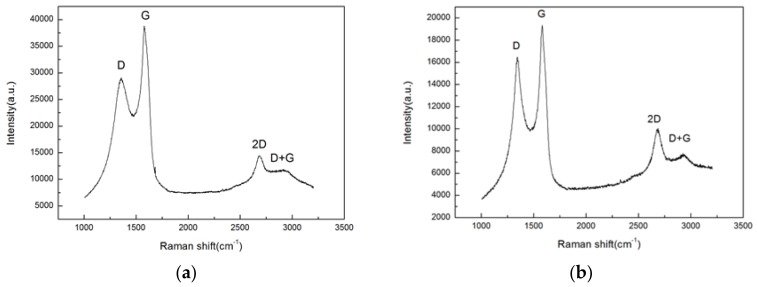
Raman results for (**a**) graphene before friction test, (**b**) the friction surfaces lubricated by the 5CB+0.15 wt.% G suspension.

Because Raman spectroscopy is a most suitable characterization technique for graphene materials [13], the Raman spectroscopy was used to further confirm that the better lubrication performance of 5CB+0.15 wt.% G is attributed to that the graphene enters the friction interface and forms a deposition layer on the friction surfaces. Figure 7a shows the Raman spectrum of the pristine graphene powders. Figure 7b shows the Raman spectrum of the wear surface of the steel plate lubricated with 5CB+0.15 wt.% G suspension. As shown in Figure 7b, the Raman spectrum of the friction surfaces lubricated with 5CB+0.15 wt.% G suspension exhibits three characteristic peaks of the G peak (1580 cm^−1^), D peak (1350 cm^−1^) and 2D peak (2712 cm^−1^) which are the typical Raman signals of graphene [14]. This directly indicates that graphene exists on the worn surfaces.

Comparing Figure 7a with Figure 7b, it can be seen that the Raman spectrum for the graphene after friction tests has some different characteristics from that for the pristine graphene powder. The intensity ratio of the I_2D_/I_G_ increased after the friction tests, which indicates that the thickness of the graphene is reduced [49,50,51]. In addition, the Raman spectrum of the graphene after the friction tests showed an increased I_D_/I_G_ ratio to 0.89, as compared with 0.75 in the pristine graphene powder. This indicates that the defect density of graphene increases after friction tests [52,53]. This result supports our previous hypothesis that graphene exists at the friction interfaces and forms a protective layer with antifriction and antiwear effects together with 5CB on the friction surfaces.

#### 3.3.4. TEM Analysis

**Figure 8:**
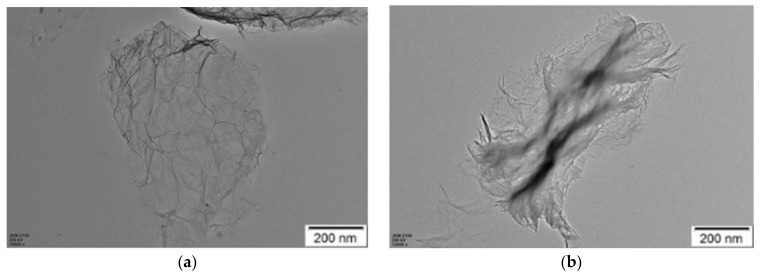
Scanning electron microscopy images of graphene: (**a**) before and (**b**) after the friction tests.

**Figure 9:**
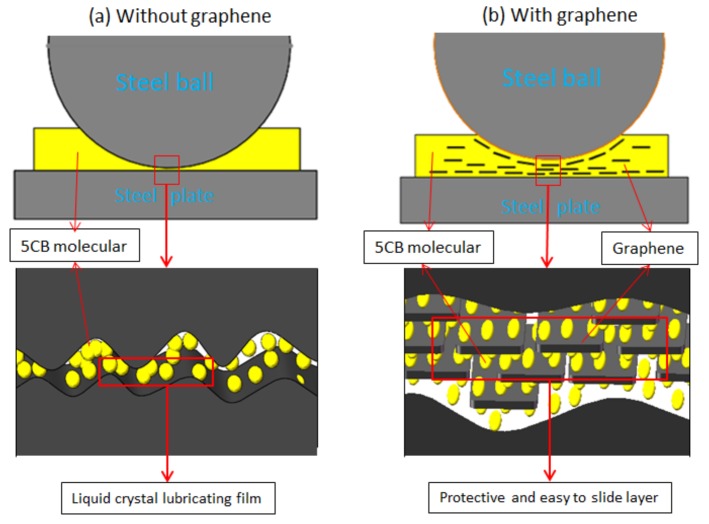
Scheme of lubrication mechanism: (**a**) Lubricated with 5CB; (**b**) lubricated with 5CB+0.15 wt.% G suspension.

Graphene protects the friction surfaces and reduces the friction coefficient by forming a protective layer on the friction surfaces during the friction process. Graphene has strong mechanical strength, so the protective layer can greatly protect the friction surfaces from abrasion and corrosion. In addition, graphene sheets with weak van der Waals force are easy to slide between each other, which helps to reduce shear resistance during friction [21,24]. In order to directly observe the changes in structure of graphene after the friction tests, TEM was used to study morphology of the graphene. The TEM morphologies of graphene before and after the friction tests were presented in Figure 8. Before the friction tests, the surface of graphene was very smooth, with a lateral size of about 0.2–2 μm as found in Figure 8a. However, after the friction tests, many folds were observed on the surface of graphene, as found in Figure 8b. This result shows that graphene bears frictional shear force in the friction tests, which changes the morphology of graphene and increases the surface defects of graphene. This result is in consistent with that of the Raman spectroscopy analysis. It further proves the lubrication mechanism that 5CB and graphene forms a protective layer on the friction surfaces to reduce friction and wear. Scheme of the lubrication mechanism is illustrated in Figure 9.

## 4. Conclusions

In this work, the lubrication performances of graphene as additive of 5CB for steel/steel contacts were studied by using a tribometer with a ball-on-plate configuration. Based on the analysis of the test results, the conclusions are as follows.

The concentration of graphene with the best antifriction and antiwear properties in the graphene/5CB suspension was 0.15 wt.%. Compared with pure 5CB, 5CB+0.15 wt.% G suspension reduced the friction coefficient and wear scar diameter by up to 70.6% and 41.3%, respectively. This outstanding lubricity is due to the low shear resistance and chemically stable protective layer formed by graphene and 5CB on the friction surfaces. Graphene with a sheet structure easily enters the friction interfaces and forms a protective layer on the friction surfaces, which makes the friction surfaces inert and prevents the two friction surfaces from direct contact, thereby reducing wear. In addition, graphene sheets are easy to slide, which is beneficial to reducing frictional resistance. Therefore, the addition of appropriate amount of graphene into 5CB can significantly reduce the friction and wear.
